# Supplementary figures and images for: CD38 promotes hematopoietic stem cell dormancy
Source: PLoS Biol. 2024 Feb 29;22(2):e3002517. doi: 10.1371/journal.pbio.3002517 (PMC10931502; doi:10.1371/journal.pbio.3002517)

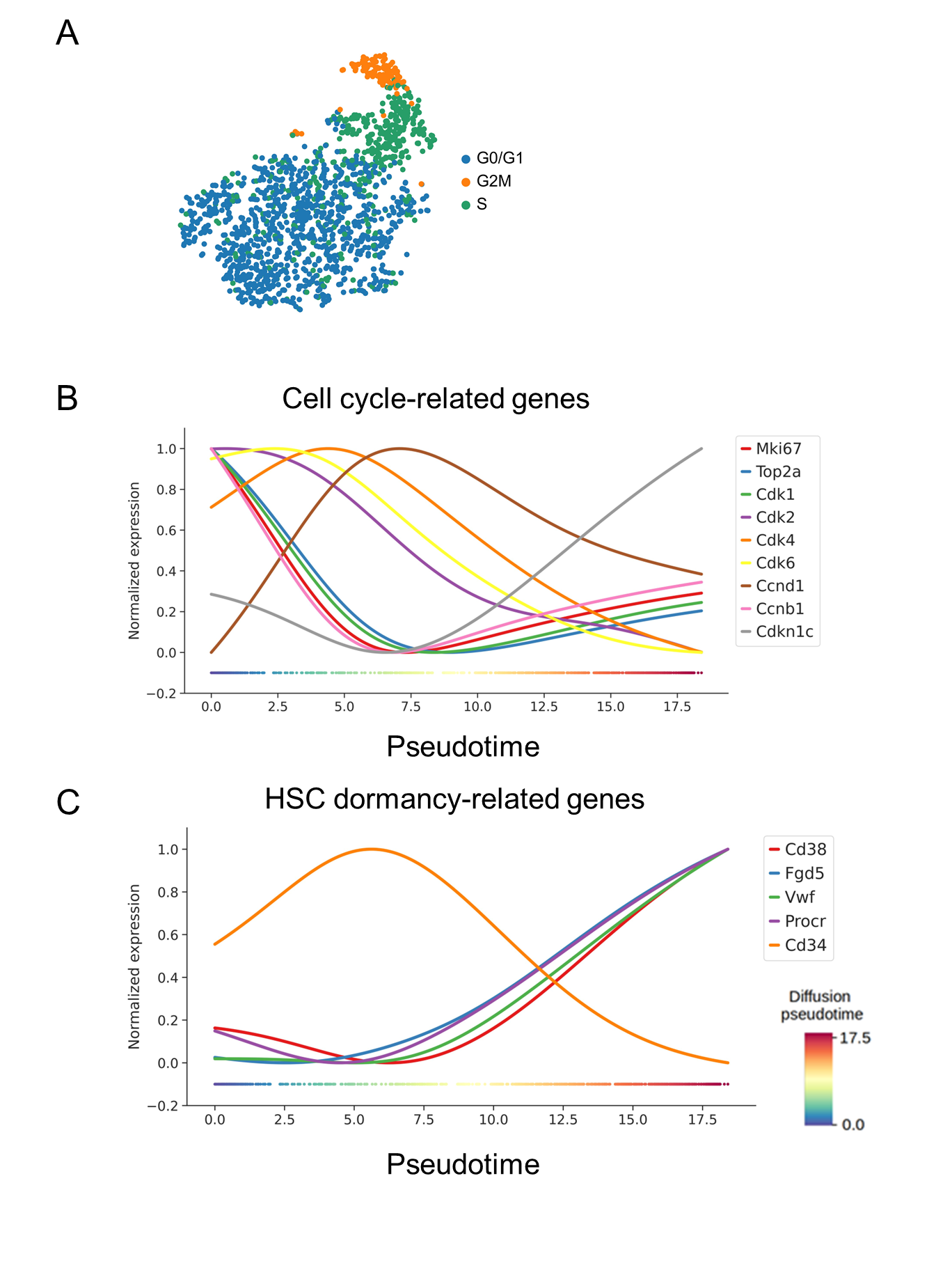

Supplement: S1 Fig — (A) Uniform manifold approximation projection (UMAP) representation depicting the transcriptional profiles of individual HSCs (LSK CD48- CD150+), clustering base on cell cycle-related genes. (B) Aligned kinetic curves for selected cell cycle-related genes along pseudotime. (C) Aligned kinetic curves for selected HSCs’ dormancy-related genes along pseudotime. The data underlying this figure can be found in S1 Table. (TIF) [file pbio.3002517.s001.TIF]

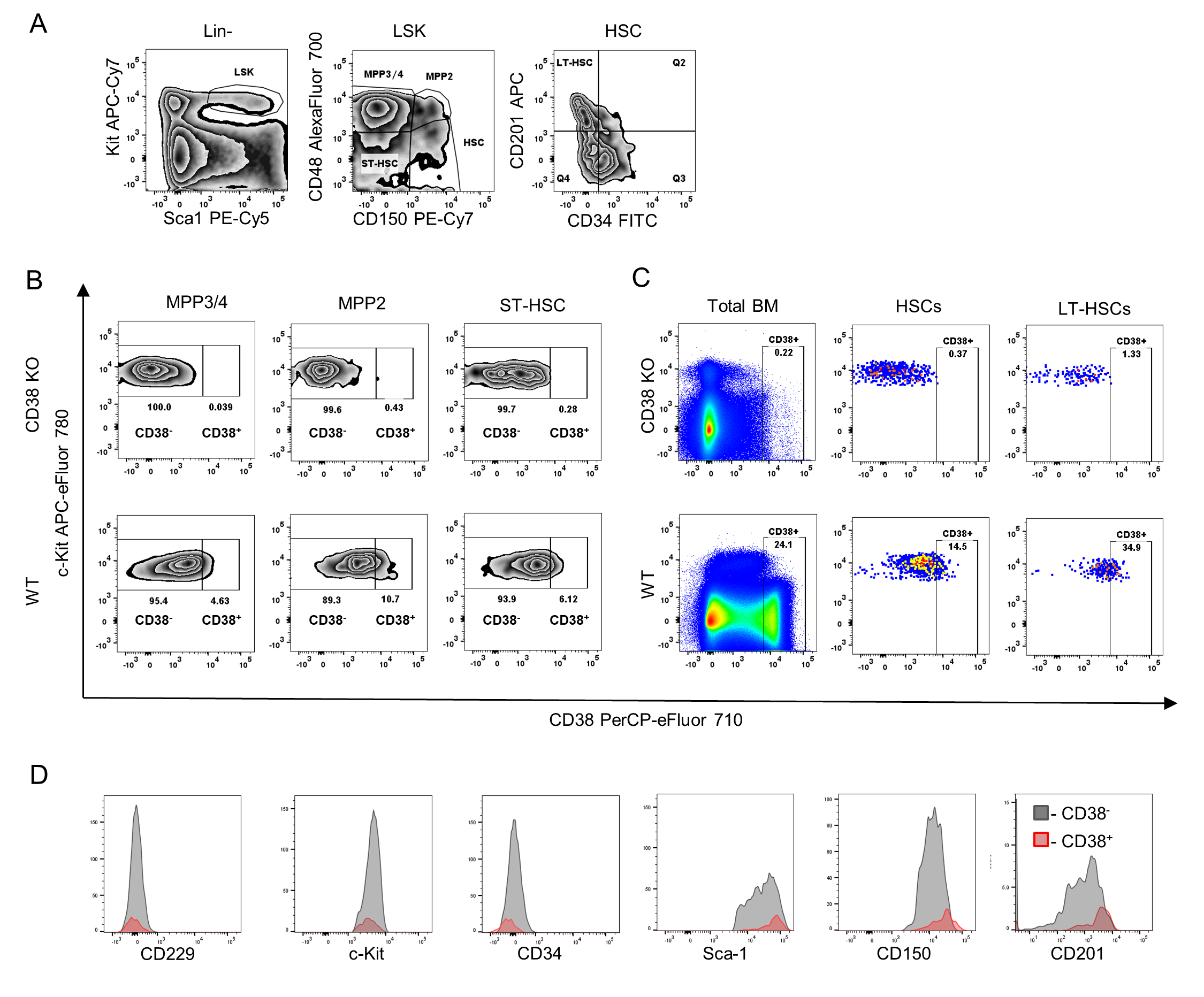

Supplement: S2 Fig — (A). Gating strategy for analysis of murine HSPCs. (B) Flow cytometry analysis of CD38 expression in HSPCs compartment (MPP3/4: Lin- Sca-1+ Kit+ (LSK) CD48+ CD150-, MPP2: LSK CD48+ CD150+, ST-HSCs: LSK CD48- CD150-, HSCs: LSK CD48- CD150+). HSPCs from CD38KO were used as negative control for gating. (C) Gating strategy for defining CD38+ fraction using total BM cells. (D) FACS analysis of defined markers surface expression on CD38- and CD38+ HSCs. (TIF) [file pbio.3002517.s002.TIF]

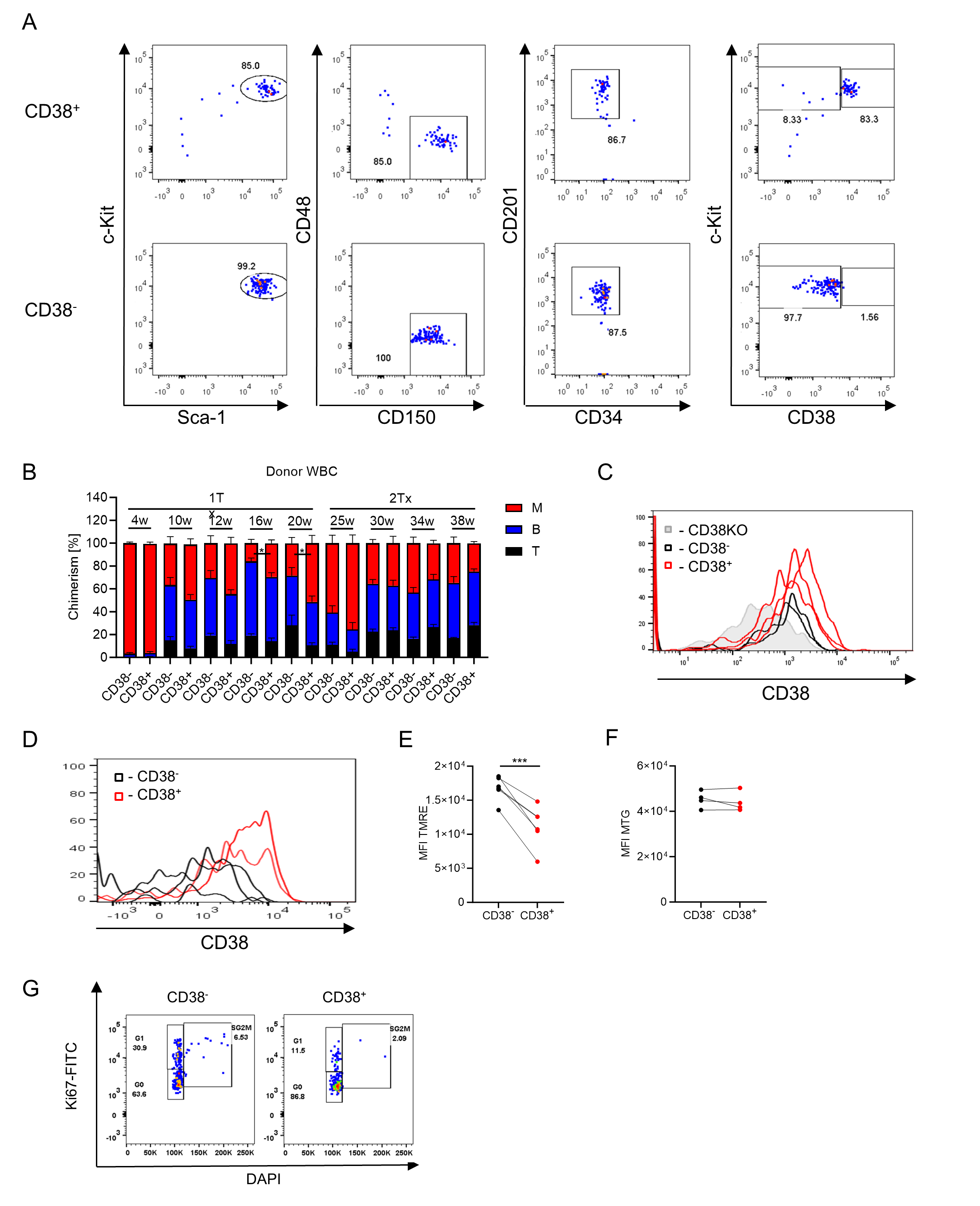

Supplement: S3 Fig — (A). Reanalysis of sorted LT-HSCs used for transplantation experiments. (B) Frequency of T, B and myeloid cells in donor-derived WBC at the different time points after primary and secondary transplantation (Fig 2D–2H). (C and D) Surface expression of CD38 in donor-derived HSCs at 20 weeks after primary transplantation of CD38+ or CD38- LT-HSCs, data from individual mice containing >60 cells in the donor HSC population in the recorded fcs file, 2 independent experiments. (E) HSC mitochondrial membrane potential analysis (n = 5). (F) Analysis of mitochondrial mass in HSCs using MitoTracker Green (MTG) in the presence of verapamil (n = 4). (G) Representative FACS plots for discrimination of G0, G1, and SG2M phases of the cell cycle of LT-HSCs. P-values were calculated using the paired t test, *** p < 0.001. The data underlying this figure can be found in S1 Data. (TIF) [file pbio.3002517.s003.TIF]

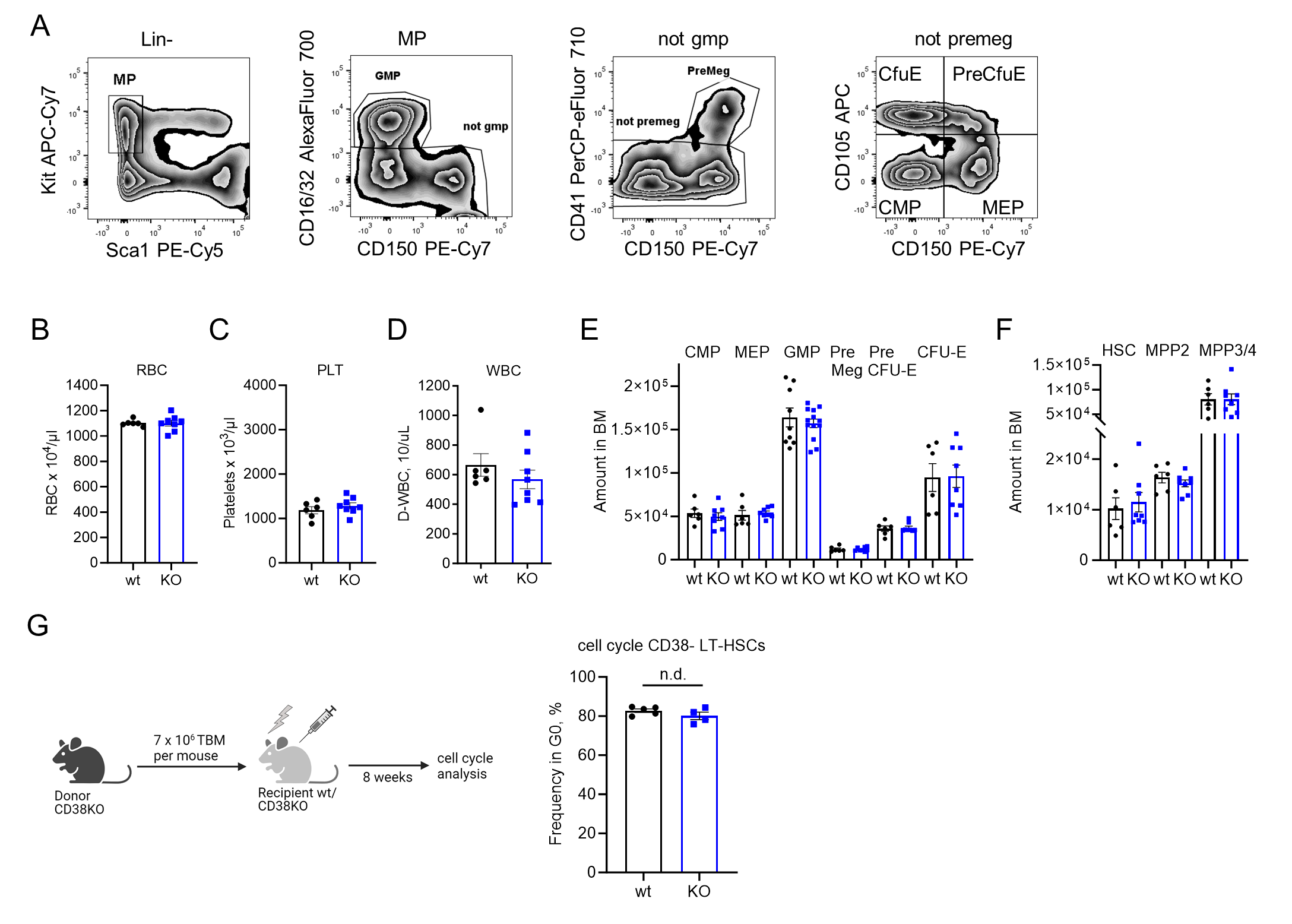

Supplement: S4 Fig — (A) Gating strategy for analysis of restricted myeloid progenitors. (B) Number of RBC in peripheral blood of wt and CD38KO (KO) mice. (C) Number of platelets (PLT) in peripheral blood of wt and CD38KO mice. (D) Number of WBC in PB of wt and CD38KO mice. (E) Number of restricted progenitors in bone marrow of wt and CD38KO mice. (F) Number of HSPCs in bone marrow of wt and CD38KO. For panels B–F, n = 6 wt, 8 KO. (G) Experimental setup for transplantation of TBM from CD38KO mice to wt and CD38KO. Created with BioRender.com. Cell cycle analysis of donor HSCs 8 weeks after transplantation using Ki67 and DAPI staining (n = 5 wt, 4 KO). P-values were calculated using Mann–Whitney test. The data underlying this figure can be found in S1 Data. (TIF) [file pbio.3002517.s004.TIF]

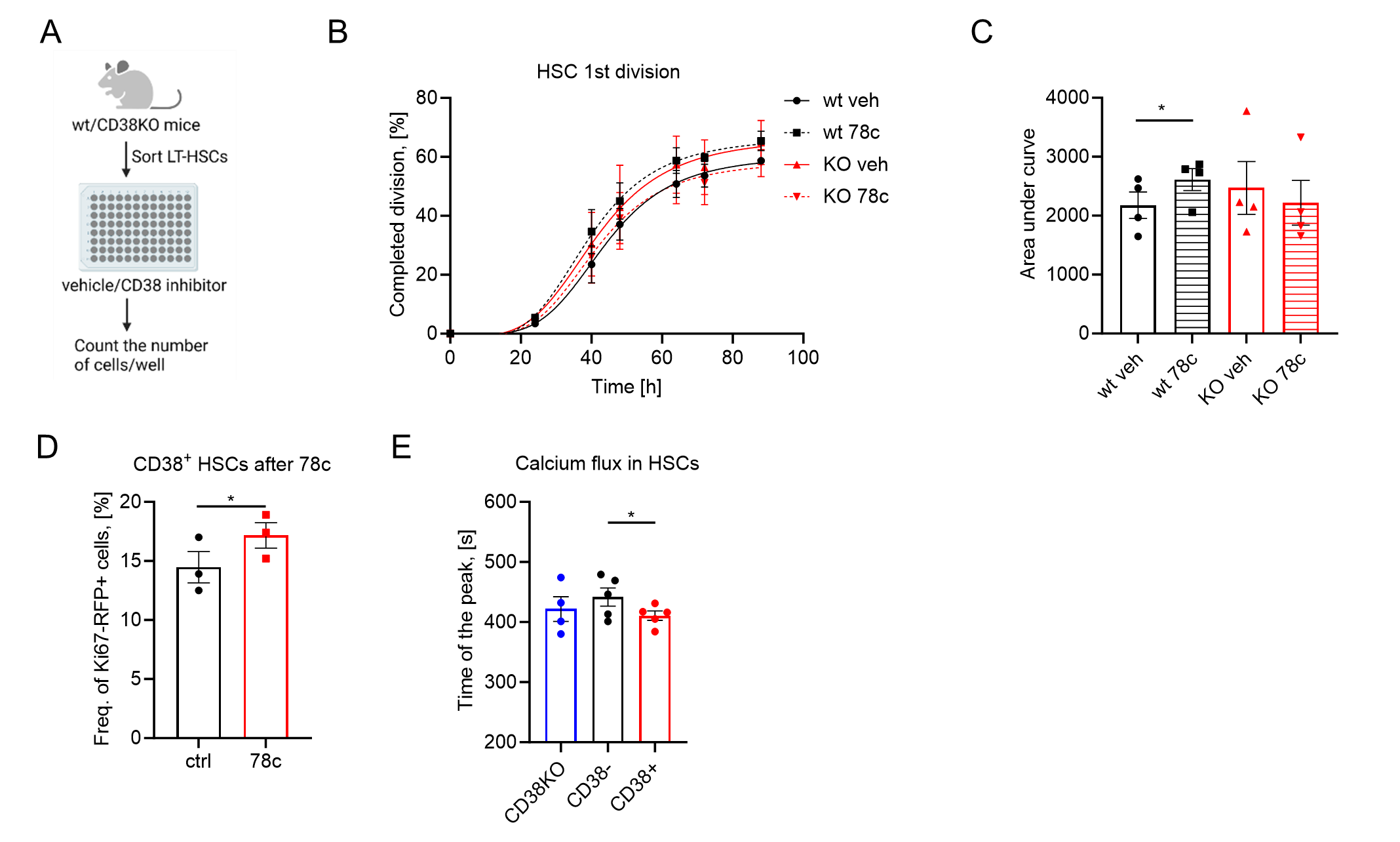

Supplement: S5 Fig — (A) Setup for single-cell division tracing experiment. Single CD38+ LT-HSCs from wt and LT-HSCs from CD38KO mice were sorted and cultured in liquid media with or without 78c. Created with BioRender.com. (B) Frequency of LT-HSCs that had completed the first division during incubation time is presented (4 independent experiments). (C) Quantification of AUC for B. (D) CD38+ HSCs from Ki67-RFP reporter mice were sorted and incubated with or without 78c (n = 3). Frequencies of RFP+ cells were analyzed 24 h later. (E) Calcium flux assay for wt and CD38KO HSCs. Time of the peak in HSC [Ca2+]c after addition of TG, n = 4 vs. 5. P-values were calculated using paired t test, *p < 0.05. The data underlying this figure can be found in S1 Data. (TIF) [file pbio.3002517.s005.TIF]

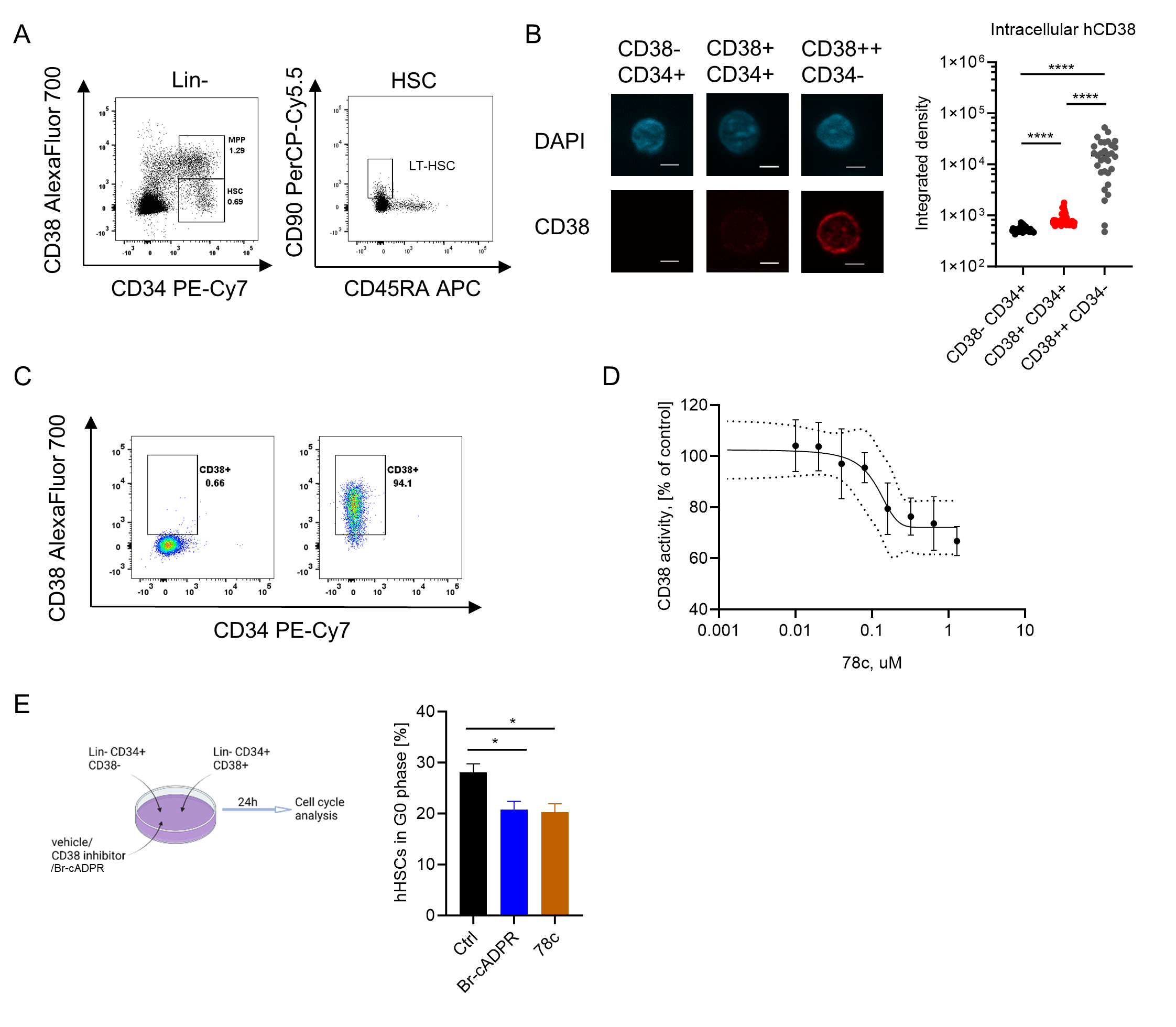

Supplement: S6 Fig — (A) Gating strategy for isolation of human HSCs and LT-HSCs. (B) Representative immunofluorescence intracellular staining of human cells and quantification of CD38 integrated fluorescence intensity (n = 43/63/31 for CD38- CD34+, CD38+ CD34+, CD38++ CD34-, respectively). (C) Surface expression of CD38 on MOLM-13. Left–isotype ctrl, right–anti-CD38 antibody. (D) Dose-response curve for cyclase activity of MOLM-13 lysates in response to CD38 inhibitor (n = 3). Sigmoidal standard curve was interpolated, 95% confidence interval is shown. (E) Lin- CD34+ human cells were sorted and incubated 24 h with or without Br-cADPR and 78c, cell cycle of CD38lo/- CD34+ cells was analyzed using Ki67 staining. Graphical scheme was created with BioRender.com. P-values were calculated using the unpaired t test, *p < 0.05. The data underlying this figure can be found in S1 Data. (TIF) [file pbio.3002517.s006.TIF]

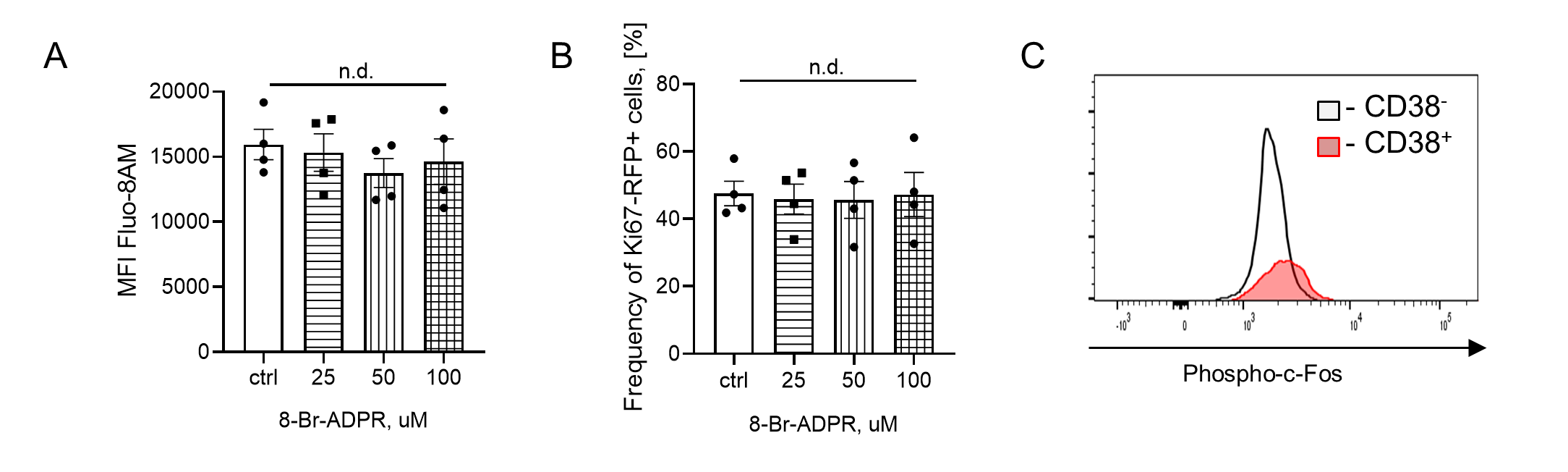

Supplement: S7 Fig — (A) LSK from Ki67RFP reporter mice were sorted and cultured for 24 h in the presence of ADPR antagonist (8-Br-ADPR). Relative [Ca2+]c concentration in HSCs treated with 0–100 μm 8-Br-ADPR, (n = 4). (B) Frequency of Ki67-RFP+ HSCs 24 h after treatment with 0–100 μm Br-cADPR, (n = 4). Multiple-group comparisons were performed using Brown–Forsythe and Welch ANOVA followed by Dunnett’s T3 multiple comparison tests. (C) Representative histogram of intracellular p-c-Fos in CD38- and CD38+ HSCs. The data underlying this figure can be found in S1 Data. (TIF) [file pbio.3002517.s007.TIF]

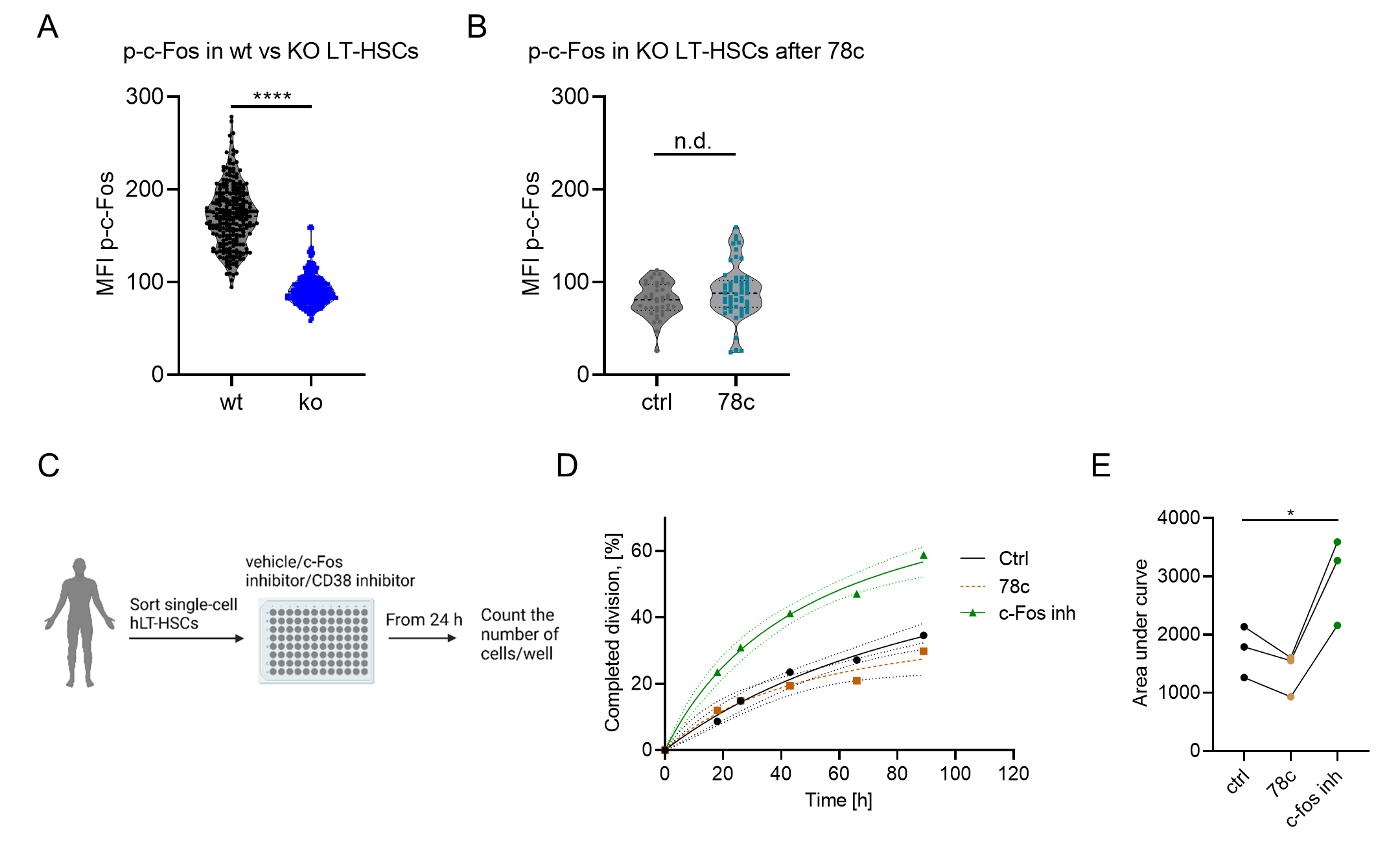

Supplement: S8 Fig — (A) Quantification of p-c-Fos MFI in LT-HSC from wt and LT-HSCs from CD38KO (n = 247—wt, n = 268—CD38KO). (B) Quantification of p-c-Fos MFI in CD38KO LT-HSC cultured for 24 h with or without 78c (n = 40—wt, n = 54—CD38KO). A, B—the P-values were calculated using unpaired t test, ****p < 0.0001. (C) Set-up of single-cell tracing experiment. Human single LT-HSCs were sorted into plate and incubated in the presence of c-Fos (T-5224) or CD38 (78c) inhibitors. Numbers of cells in wells were detected. Created with BioRender.com. (D) Frequency of LT-HSCs that had completed the first division during incubation time is presented for 1 donor. (E) Quantification of the area under the curve (AUC) for data in panel D for 3 donors. Multiple-group comparison was performed using Brown–Forsythe and Welch ANOVA followed by Dunnett’s T3 multiple comparison tests. The data underlying this figure can be found in S1 Data. (TIF) [file pbio.3002517.s008.TIF]
