# Supplementary material for: CD38 promotes hematopoietic stem cell dormancy
Source: PLoS Biol. 2024 Feb 29;22(2):e3002517. doi: 10.1371/journal.pbio.3002517 (PMC10931502; doi:10.1371/journal.pbio.3002517)
Supplement: Attachment — Submitted filename: rebuttal_letter_Ibneeva_et_al.pdf [file pbio.3002517.s016.pdf]

The authors would like to thank the reviewers for their valuable comments and suggestions. We have carefully considered all of the points raised and revised our manuscript accordingly and added new data to support our finding. In the rebuttal letter below, we have extensively discussed all the different concerns and adjustments we made to our work. The reviewers' comments are in blue and the authors' responses are in black.

REVIEWS:

Reviewer #1: In this study, Ibneeva et al demonstrate a role for CD38 both in defining the dormant subset of murine LT-HSC, and functionally in maintenance of HSC dormancy through an axis that involves CD38 enzymatic activation of cADPR, Ca2+ flux, and downstream cFos-mediated expression of the cell cycle regulator, p57Kip2. Overall, the experiments provide strong evidence for this mechanism by leveraging multiple complementary techniques and robust serial transplantation assays. The manuscript is well-written, easy to follow, and concise. The findings are of significant impact by providing novel mechanistic insight into the regulation of HSC dormancy, linking complementary findings from previously published manuscripts, as well as identification of a novel surface marker for the most dormant subset of murine LT-HSC. Although some evidence is provided to suggest a similar (non-cell autonomous) role for CD38 in human HSCs, the precise role in human HSC dormancy remains to be shown conclusively through functional analysis. However, given the breadth of the current study, I do not feel additional human HSC experiments are necessarily in scope, but would be intriguing to study further in future studies. Overall, I believe the manuscript warrants publication in PLOS Bio as it may be of broad interest to the hematopoiesis and stem cell research communities.

I have only several minor comments that should be addressed prior to publication:

1) Line 85: Sorting/gating strategy for LT-HSCs used for scRNAseq should be included in the data in Fig 1 or as a supplemental figure. If obtained, post-sort purity checks should also be shown

For scRNA sequencing we sorted HSCs (LSK CD48- CD150+). Gating/sorting strategy is shown in Fig. S 2A. Post-sort purity was not obtained.

2) Figure 1 should include how cells clustered in UMAP space, or this should be included in the supplement, for reference.

We have shown clustering of cells in UMAP space in Fig. 1A and added a new clustering based on cell cycle related genes (S1 Table) to Fig. S 1A.

Cells were clustered using ReduceDimension() and clusterCells() functions by Monocle 2. ReduceDimension() function is run to perform tSNE dimensionality reduction method using these parameters: *num_dim = 10*, *norm_method = 'log'*. For unsupervised clustering of cells, we used clusterCells() function. We added detailed information to Material and Method section Lines: 541-542.

3) Figure 1A: How was pseudotime ordering determined? The root of pseudotime is often chosen arbitrarily. If that is the case, it might be more intuitive to choose the dormant HSC state as the root.

For our analysis, we chose to order cells in an unbiased way because it is the first step for 'orderCells' function, but choosing a dormant HSC resulted in similar pseudotime trajectory as well. Also, dimensionality reduction methods mostly employ unbiased clustering, so we chose pseudotime root to be unbiased.

4) Fig 2D: Post-sort purity checks by FACS, if performed, should be shown for all sorting experiments to assess purity of sorted populations for transplant.

We added post-sort purity checks for transplanted populations in Fig. S 3A.

5) Line 220: Please explain what is the putative neighboring CD38+ cells in the context of hHSC in the marrow niche in vivo. Are these proposed to be stromal populations, or more differentiated hematopoietic progenitors?

Different populations of cells in the human bone marrow have been reported to express CD38: multipotent and restricted hematopoietic progenitors, plasma cells, activated T and B-lymphocytes, and NK cells [1]. Therefore, some of these CD38+ cells can be the neighbors for hHSCs.

We added this information to Line: 466-468.

6) Figure 4I: These experiments should also be performed in the absence of CD38+ MOLM13 cells, to show that the 78c inhibitor has no effect on cell cycle in the absence of paracrine CD38-

expressing cells. Also were MOLM13 lysates sufficient to affect cell cycle in these assays (since Fig S4 shows CD38 activity presence in the MOLM13 lysates that could be inhibited by 78c)?

We treated $CD38^{lo/-}$ hHSCs cells with the 78c inhibitor and as expected we found that inhibition of CD38 enzymatic activity did not influence the proliferation of human CD38lo/- HSCs in the absence of CD38+ cells. We added this experiment to Fig. S6E.

While we appreciate the suggestion of the reviewer, we believe that adding MOLM-13 lysates to hHSC culture per se may affect their cell cycle, as cell lysates will contain DNA, RNA and proteins from MOLM-13 cells, which can lead to unspecific hHSC activation. Therefore, it would be difficult to elucidate the inhibitory role of CD38 on hHSC proliferation in such experimental setting.

7) Figure 3: Human LT-HSCs are more highly purified within the CD34+CD38-/lo population based on expression of additional markers (such as CD90, CD49f, CD201). The authors should comment on the limitations of the analysis in figure 3 in that the CD34+38-/lo population is likely to be heterogeneous in regards to dormancy.

We agree with the reviewer that the population of CD34+CD38-/lo cells is heterogeneous; however, the goal of the experiment was to show that CD38+ neighboring cells could regulate the cell cycle activity of CD38lo/- HSCs. We added information about the gating strategy in Lines: 251-253.

8) Figure 4G: Did [Ca2+]c also increase significantly faster in CD38 wt compared with CD38 KO LT-HSCs, as seen for CD38+ vs CD38- cells?

We have not observed a significant difference in calcium accumulation time between CD38+ and CD38KO HSCs (Fig. S5E). This could be due to compensatory mechanisms at the organismal level in CD38KO mice. Previous study has reported that CD38KO mice are protected against glucose intolerance induced by a high-fat diet and have increased energy expenditure under homeostatic conditions, suggesting large differences in metabolism between wt and CD38KO mice [2]. We added information in Lines: 293-298.

9) Figure 6: Did Br-cADPR treatment also decrease p57kip2 MFI?

Indeed, treatment of HSCs with Br-cADPR decreased the p57kip2 MFI. We added this data in

Fig. 8I.

Reviewer #2, Tsuyoshi Fukushima (note, Reviewer 2 has signed this review): This is an interesting paper showing that cytoplasmic calcium may be involved in HSC dormancy and the possibility that this regulation is mediated by CD38, cADPR, Fos, p57. Overall, the paper would be better if more is learned about the importance of CD38/cytoplasmic culcium to the physiological significance.

We thank the reviewer for the suggestion and analyzed the activation of CD38+ and CD38- LT-HSCs in response to different stress conditions (added as a new Fig. 3F-G).

major coment

① CD38 has a similar behavioral expression as other stem cell markers. Sorting for CD38+ will sort fractions with high levels of these stem cell markers. This means that the comparison of CD38+ and CD38- indicates that HSCs are enriched in the CD38+ fraction.

Therefore, it is necessary to verify whether CD38 is important for dormancy and stemness in the CD38 KO mice.

It would be important to analysis cell cycle, label retaining, evaluation of stress systems such as LPS or 5FU, and aging in the CD38 KO mice.

We agree with the reviewer that if we analyzed the population of HSCs (LSK CD48- CD150+) CD38+ vs CD38-, CD38+ HSCs will be enriched with long-term hematopoietic stem cells (LT-HSCs). However, to investigate the role CD38 in the behavior of LT-HSCs, we sorted the same amount of LT-HSCs with the phenotype: LSK CD48- CD150+ **CD201+ CD34-** CD38+ vs CD38- and demonstrated their superior stem cell properties in a competitive serial transplantation assay (Fig. 2E-G). In Fig. 3A-E and new Fig. 3F-G, we analyzed the cell cycle of LT-HSC CD38+ vs. CD38- to avoid the difference in the population quiescence due to different rates of HSC enrichment.

Furthermore, we have shown that in competitive serial transplantation assay, CD38KO LT-HSCs and CD38KO TBM have decreased repopulation capacity compared to wt cells.

In addition, we compared the cell cycle of CD38KO and wt LT-HSCs (new Fig. 4K-L added). CD38KO LT-HSCs were less quiescent compared to their CD38+ counterparts. However, CD38- LT-HSCs were less quiescent than CD38KO cells. This could be due to compensatory mechanisms adopted at the organismal level in CD38KO mice. Previous study has reported that CD38KO mice are protected against glucose intolerance induced by a high-fat diet and have increased energy expenditure in homeostatic conditions, implying large differences in metabolism between wt and CD38KO mice [2]. Additionally, CD38 is expressed on various immune cells, such as B-cells, T-cells [3], and macrophages [4, 5]. Hence, indirect effects of the CD38 deletion in these cell populations could affect HSC quiescence and function in CD38KO mice. We added new experiments in Lines: 215-218, and in Fig. 4K-L.

Therefore, comparing CD38- and CD38+ LT-HSCs from the same mouse is a more relevant way to analyze the behavior of two cell types under different conditions. We added new experiments investigating the cell cycle entry of CD38+ LT-HSCs in response to hematopoietic stressors: polyI:polyC (pIC) mimicking viral infection, or 5-fluorouracil (5-FU), a myeloablative agent. We found that while CD38- LT-HSC rapidly entered the cell cycle in response to pIC, fewer CD38+ LT-HSCs entered the cell cycle and did so with a significant delay (Fig. 3F). In contrast, both CD38- and CD38+ LT-HSCs actively proliferated 4 days after 5-FU injection (Fig. 3G). Although CD38+ LT-HSCs tended to restore their quiescence 8 days after 5-FU injection, CD38- cells remained in the cell cycle. Thus, as CD38+ LT-HSCs required more time to enter the cell cycle in response to hematological stresses and returned faster to quiescence compared to CD38- cells, we posit that high levels of CD38 expression define LT-HSCs in deep in state of quiescence (dormant LT-HSCs) not only in steady state but also under hematopoietic stress. We added new experiments in Lines: 186-195, and in Fig. 3F-G.

② To determine the extent how environmental CD38 is affecting HSCs, CD38KO HSC can be transplantated into CD38KO and WT recipients.

Also, if there is a difference in this experiment, To explain the degree of dependence of the environment and the HSCs themselves on CD38 by transplanting CD38 KO and WT HSCs to CD38 KO and WT rescipient.

We transplanted CD38KO cells into CD38KO and WT recipients and demonstrated that CD38 from the BM microenvironment did not influence the cell cycle of CD38KO HSCs (Fig. S4G). Therefore, the surface expression of CD38 on mouse HSCs plays a detrimental role in their quiescence regulation. We added the data into Fig. S4G, Lines: 218-220.

③ In humans, can you explain the selectivity for HSCs, why HSCs specifically are dormant when cADPR is of environmental origin?

It was outside the scope of the current study to investigate whether quiescence of other cells is dependent on the distance from CD38+ neighbors. However, we defined that c-Fos promotes the quiescence of murine and human LT-HSCs (new Fig. S8C-D added). The transcription factor c-Fos does not work alone but in a complex with other proteins (e.g. c-Jun, JunB, Fra1, and others proteins from the AP1 complexes) [6]. Therefore, we suggest that the specific partner in different cell types could determine the cell type specific transcriptional profile and cell response to environmental cADPR, therefore this possibility should be investigated in future research.

The importance of the production of cADPR by HSCs themselves in humans can be evaluated by analysis of cell cycle or division time by addition of 8-Br-cADPR.

Indeed, the treatment of hHSC with 8-Br-cADPR activates the cell cycle of hHSC. We added this data into Figure S6E.

If there is a difference in the above results, CD38 may not be on the surface in humans, but may be present intracellularly. It may be in the endoplasmic reticulum, nuclear membrane, or lysosomes, but cADPR could still be produced. It might be interesting to look at localization to know the possibilities. There may also be the possibility of other ADPR cyclases such as CD157 (BST1).

We thank the reviewer for this very relevant question. We analyzed the intracellular CD38 in CD38lo/- human HSCs and found that low surface expression of CD38 correlates with low intracellular CD38 levels Fig. S6B. Indeed, the role of another ADPR cyclase (CD157) in the regulation of quiescence is very interesting and should be investigated in the future.

④ Is the Fos is activated in CD38+ also seen in RNAseq? For example, activation of AP1target, gene with AP1 motif, etc.
(Figure 5D is a gene with a cFos motif in a CD38+ gene with high expression. So you should check GSEA or GO etc for cFos motif gene etc.)

Indeed, in the GSEA, we found an enrichment of AP-1 responsive genes in CD38+ LT-HSC (we added a new Fig. 7F), Lines 339-340. Moreover, several significantly upregulated genes in CD38+ LT-HSC have c-Fos motifs up-stream of their promoter region (S5 Table in text).

**Name**	**start**	**end**	**pvalue**	**matched_sequence**
H19	534	541	6.10E-05	ATGAGTCA
Clu	142	149	3.05E-05	GTGACTCA
Cd38	7	14	1.53E-05	GTGAGTCA
Cd38	1086	1093	6.10E-05	ATGAGTCA
Gprc5c	2338	2345	7.63E-05	GTGATTCA
Cdkn1c	522	529	3.05E-05	GTGACTCA
Cdkn1c	1999	2006	3.05E-05	GTGACTCA

Also, does the 78c and 8-Br-cADPR treat decrease Fos target gene expression (including p57)?

Indeed, 78c and 8-Br-cADPR treatments lead to a decrease of p57kip2 (Fig. 8K + we added new Fig. 8I)

minor coment
①It is easier to understand if the Psudo time scale is reversed.

We changed the pseudotime scale in Figure 1.

② In vivo administration of BrdU for more than 1 day seems to induce cytotoxicity and stress responce. Differences in responsiveness to stress may be appreciated, but should be evaluated with caution.

We added new experiments investigating the cell cycle entry of CD38+ LT-HSCs in response to hematopoietic stressors: polyI:polyC (pIC) mimicking viral infection, or 5-fluorouracil (5-FU), a myeloablative agent. We found that CD38+ LT-HSCs required more time to enter the cell cycle in response to hematological stresses and returned faster to quiescence compared to CD38- cells, we posit that high levels of CD38 expression define LT-HSCs, which are more deeply in a state of quiescence not only in steady state but also under hematopoietic stress (Fig 3F, G).

③ Data from CD38 KO transplantation evaluated the importance of the CD38/calcium pathway in hematopoietic stem cells. This data is so important to this paper that it should be a main Figure.

We moved this data to the main figures (Fig. 4 A-J).

Reviewer #3: Ibneeva et al., identify CD38 as a marker of dormant HSCs initially via transcriptomic profiling of single HSCs. They further show that the CD38 is enzymatically active on HSCs. The authors attempt to demonstrate that CD38 regulates cFos levels and p57kip2 protein via cFos in dormant HSCs. Although the data on CD38 is interesting, the data on CD38 regulation of cFos and p57 is greatly limited and preliminary. My specific comments are below:

1) It is unclear and confusing how the initial analysis was performed; it should be clarified: "We observed that cells in the S (Fig. 1B) and the G2/M phases (Fig. 1C) were clustered together and that, as expected, most of the HSCs were quiescent (Fig. 1D)". Did the authors determine HSCs in S, G2M and G0 based on Seurat analysis? Did they compare the bioinformatic analysis to functional properties?

Indeed, we used Seurat analysis to identify the cell cycle score, as written in Lines 90-91: ‘To identify actively cycling cells, we calculated cell cycle and dormancy scores of individual HSCs

using Seurat [12], which were based on the expression levels of cell cycle and dormancy genes [13] (S1 Table).

Further, we compared the bioinformatics analysis with functional properties of CD38+ HSCs: Fig. 2A-I and Fig. 3A-G.

1. We compared the expression of CD38 and other LT-HSC related markers: (Fig. 2A-C, Fig. S2D) and have shown the enrichment of CD201+, CD34- cells in CD38+ HSC population compared to CD38- cells. In addition, we found that the surface expressions of CD229 and c-Kit were lower, while those of Sca-1, CD150 were higher in CD38+ HSCs (Fig. S2D). Taken together, these data indicate that CD38+ HSCs exhibit a phenotype of the most potent and quiescent LT-HSCs.

2. We performed cell cycle analysis using Ki67 and DAPI staining, BrdU incorporation, in vivo label retention assay, single cell tracing experiment to confirm quiescence of CD38+ LT-HSC (Fig. 3A-G, Fig. 5A-C). We have shown that CD38+ LT-HSCs are deeper in the state of quiescence than CD38- cells.

3. We compared the repopulation capacity of CD38+ vs CD38- LT-HSCs in Figure 2D-I. CD38+ LT-HSCs have higher repopulation and self-renewal capacity compared to CD38- LT-HSCs.

Therefore, using multiple functional assays, we confirmed our hypothesis from single cell transcriptomic analysis that CD38 can be used to enrich LT-HSCs that are in the deep quiescence state and have high repopulation capacity.

2) The authors state: "Notably, this cluster was characterized by genes involved in pathways related
to the activation of tumor necrosis factor alpha (TNFα) signaling, interferon gamma and alpha response, Stat3 and Stat5, as well as transforming growth factor beta 1 (TGF-β1) signaling, which is a well-known regulator of HCS quiescence". This cluster seems to be a collection of genes important for both HSC quiescence and activation.

The authors thank the reviewer for this interesting question. Indeed, we found the up-regulation of interferon gamma and alpha response in the cluster associated with quiescent state as well as with markers defining the most functional HSCs (Fgd5, Procr, and vWF). Our results are in agreement with the previous publication [7], which suggests that intrinsic expression of interferon responsive genes in HSCs in a steady-state condition confers antiviral resistance to

adult HSCs, similar to what has been shown for human fetal HSCs and other stem cell types [8]. The upregulation of TNFa signaling in this cluster is in agreement with other finding suggesting the pro-survival role of TNF a signaling in HSCs [9]. The up-regulation of Stat5 signaling in the most functional HSCs is consistent with previous publications showing the important role of Stat5 in HSC self-renewal [10] and quiescence support [11]. The importance of Stat3 signaling for HSC functionality (regulation of self-renewal, apoptosis, DNA repair, ROS and internal IFN signaling) has been shown in other recent work [12].

We added this info into the discussion Lines: 406-419.

3) The data presented using available assays such as BrdU and analyses of H2BGFP suggest that CD38+ LT-HSCs are enriched for dormant HSCs but not that they are dormant HSCs. I would suggest instead of labeling "CD38+ LT-HSCs as dHSCs" , describing CD38+LT-HSCs as relatively enriched in dormant HSCs as compared to CD38- LT-HSCs.

We agree with the reviewer and changed our statement in Line 195.

We also changed the labeling of dHSCs to CD38+ LT-HSCs throughout the entire paper.

4) Experiments using 78c would benefit from using CD38 KO HSCs.

We agree with the reviewer and added data to the manuscript.

In Fig. S4A, we have shown that 78c did not affect the exit from quiescence of CD38KO LT-HSCs, while activated wt LT-HSCs. We have shown that inhibition of CD38 enzymatic activity did not influence the levels of p-c-Fos and p57Kip2 in CD38KO HSCs (Fig. S8B, Fig. 8K), while decreased the level of p-c-Fos and p57Kip2 in wt cells (Fig. 8F, Fig. 8K).

5) The statement "and high cytoplasmic Ca2+ has been shown to support quiescence of HSCs" and the Fukushima et al., publication that the authors cite is debated as two other publications (PMID: 29946000; PMID: 31178255) that the authors did not cite, show that increased Calcium triggers HSCs activation. The authors should be cautious and discuss fully their results in the context of all previously published results and not a selected subgroup.

We agree with the reviewer that all previous findings should be valued. In the study by Luchsinger and colleagues [13], $[Ca^{2+}]_c^{low}$ HSCs displayed enhanced long-term repopulation capacity compared to $[Ca^{2+}]_c^{high}$ HSCs. However, the conclusion from this work appears contradictory when considering previous research from this group. In the earlier work, they suggested that HSC with higher CD150 protein levels are associated with higher $[Ca^{2+}]_c$ [14], whereas it was shown that $CD150^{hi}$ HSC have superior repopulation capacity compared to $CD150^{neg}$ HSCs [14]. However, when only dHSCs and not bulk HSCs were sorted into two groups for transplantation based on their $[Ca2+]_c$ levels, an association between higher $[Ca2+]_c$ and increased dHSC reconstitution potential was uncovered [15]. Intriguingly, in 2018 Umemoto et al. showed that elevating $[Ca^{2+}]_c$ in HSCs is important for the late G1 phase cell cycle phase under stress induced by 5-FU or LPS [15-17]. Therefore, high $[Ca^{2+}]_c$ may serve different roles across different cell cycle phases probably due to the different $[Ca^{2+}]_c$ threshold levels. Taken together, our results along with these findings suggest that while high $[Ca^{2+}]_c$ keeps HSCs dormant in the quiescent phase, higher $[Ca^{2+}]_c$ prompts HSCs to progress through the cell cycle phases. In conclusion, it is of great interest to the field to study how Ca^{2+} regulates fate decisions of dHSCs in future research.

We added this information in the discussion section Lines: 434-454.

6) It is intriguing that the authors do not see differences in CDK4/6 between CD38+ and CD38- HSCs; are there discrepancies between Fig 1G and Fig 5G? Have the authors considered that CD38+ cells may be an intermediate population between deep HSC dormancy to activation?

In Fig. 1G, we analyzed HSCs (LSK CD48- CD150+), whereas in Fig. 5G we investigated LT-HSCs (LSK CD48- CD150+ CD34- CD201+). The latter are a more quiescent population and express the Cdk4/6 at lower levels than HSCs [7]. Considering that CD38+ LT-HSCs express Gprc5c, the proposed marker for dHSCs [7], we conclude that the CD38+ LT-HSC population is enriched in dHSCs compared to the CD38- population.

7) The data on CD38 regulating cFos and p57 is preliminary, indirect and relatively weak. It might be better to move this data as supportive finding to the Supplement and modify the related statements.

While we appreciate the reviewer's suggestion, we respectfully disagree. We found that CD38+ LT-HSCs expressed significantly higher levels of c-Fos and p57Kip2 at the mRNA and protein

levels (Fig. 7A, D, E, Fig. 8A, H). Gene set enrichment analysis (GSEA) revealed a significant up-regulation of genes related to AP-1 complex in CD38+ LT-HSCs compared to CD38- (Fig. 7F). We have shown that CD38 cyclase activity is responsible for the high cytoplasmic Ca2+ levels (6D, G, H, I). Treatment of HSCs with a cADPR antagonist (Br-cADPR) reduced the levels of active p-c-Fos and p57Kip2 (Fig. 8G, Fig. 8I). We added new data showing that consistent with our hypothesis, CD38 regulates levels of c-Fos and p57Kip2 in HSCs. Furthermore, we found that their levels were significantly higher in $CD38^{+}$ wt HSCs compared to CD38KO cells (Fig. 8K, Fig. S 8A). In line with these data, inhibition of CD38 enzymatic activity decreased the levels of p-c-Fos (Fig. 8F, Fig. 8K) and p57Kip2 in CD38+ LT-HSC but not in CD38KO cells (Fig. 8K, Fig. S8B). Therefore, we believe that our data support the notion that the CD38/cADPR/Ca^{2+} axis regulates c-Fos and p57Kip2 levels in $CD38^{+}$ LT-HSCs.

8) The title: "CD38 promotes HSC dormancy" is an overstatement; there is very limited data/if any in this work suggesting "promoting HSC dormancy". The data show that "CD38 enriches for dormant HSCs".

We understand the reviewer's suggestion, but we believe that the large number of experiments we performed directly support the conclusion that CD38 does indeed promote LT-HSC the state of deep quiescence. We have shown in the single cell tracing experiment (which is well accepted in the field [7, 18] that blocking the enzymatic activity of CD38 leads to the activation of cell cycle (new Fig. S5D) and cell divisions, exclusively in $CD38^{+}$ LT-HSCs but not in $CD38^{-}$ and CD38KO cells (Fig. 5A-C). Moreover, inhibition of CD38 in vivo delayed the return of CD38+ HSCs to quiescence after 5FU treatment (Fig. 5D). In addition, we have demonstrated that inhibition of the CD38 enzymatic activity leads to the reduction of p57kip2, a well-known and essential cell cycle inhibitor of HSCs [19]. Therefore, we conclude that our data collectively demonstrate that CD38 can promote HSC quiescence.

9) The abstract should be modified accordingly.

We thank the reviewer for the suggestion and added the statement into the abstract (Line 42-44):

Here, we identify CD38 as a novel and broadly applicable surface marker for the enrichment of murine dHSCs.

10) The authors should consider replacing the statement "more quiescent" by "more deeply in a state of quiescence".

We replaced statement in Line: 181.

List of changes:

1. We have reversed the direction of pseudotime at the Fig. 1 as was suggested by two reviewers.
2. We have moved transplantation experiments from Fig. S3 to main Fig. 4 as suggested by 2nd reviewer.
3. We have moved Fig S4H to Fig. 5F.

New experiments

1. Fig. 3F, G: cell cycle analysis after in vivo treatment with pIC and 5FU.
2. Fig. 4K, L: cell cycle analysis of CD38KO and wt LT-HSCs.
3. Fig. 5D: cell cycle analysis of LT-HSCs after in vivo treatment with 5FU and CD38 inhibitor (78c).
4. Fig. 7F: GSEA enrichment of AP-1 responsive genes in CD38+ LT-HSC compared to CD38- counterparts.
5. Fig. 8F: p-c-Fos MFI in HSC after CD38 inhibitor treatment.
6. Fig. 8I: p57Kip2 MFI after treatment of HSC with Br-cADPR.
7. Fig. S 1A UMAP clustering of HSCs based on the expression of cell cycle-related genes.
8. Fig. S 3A: purity analysis of sorted LT-HSCs before transplantation.
9. Fig. S 3G: Representative FACS plots for discrimination of G0, G1 and SG2M phases of the cell cycle of LT-HSCs.
10. Fig S 4G: cell cycle analysis of donor CD38KO HSCs eight weeks after transplantation into CD38KO and wt recipients.
11. Fig. S 5D cell cycle activation of $CD38^+$ LT-HSCs after 78c treatment.
12. Fig S 5E: comparison of Ca flux in CD38KO and wt HSCs.

13. Fig. S 6A: gating strategy for hLT-HSCs.
14. Fig. S 6B: intracellular immunofluorescence staining of CD38 in CD38lo/- hHSCs and CD38+ HSPCs and BM cells.
15. Fig. S 6E: cell cycle analysis of hHSC after incubation in the presence of CD38+ HSPCs with Br-cADPR and CD38 inhibitor (78c).
16. Fig. S 8A: comparison of p-c-Fos MFI in $CD38^+$ and CD38KO LT-HSCs.
17. Fig. S 8B: comparison of p-c-Fos MFI in CD38KO LT-HSC after treatment with vehicle or 78c.
18. Fig. S 8C-E: single cell tracing experiment of hLT-HSC with 78c and T-5224 treatment.

References

1. Hogan KA, Chini CCS, Chini EN. The Multi-faceted Ecto-enzyme CD38: Roles in Immunomodulation, Cancer, Aging, and Metabolic Diseases. Front Immunol. 2019;10:1187. Epub 2019/06/20. doi: 10.3389/fimmu.2019.01187. PubMed PMID: 31214171; PubMed Central PMCID: PMCPMC6555258.

2. Barbosa MT, Soares SM, Novak CM, Sinclair D, Levine JA, Aksoy P, et al. The enzyme CD38 (a NAD glycohydrolase, EC 3.2.2.5) is necessary for the development of diet-induced obesity. FASEB J. 2007;21(13):3629-39. Epub 20070621. doi: **10.1096/fj.07-8290com**. PubMed PMID: 17585054.

3. Zeidler JD, Chini CCS, Kanamori KS, Kashyap S, Espindola-Netto JM, Thompson K, et al. Endogenous metabolism in endothelial and immune cells generates most of the tissue vitamin B3 (nicotinamide). iScience. 2022;25(11):105431. Epub 20221023. doi: 10.1016/j.isci.2022.105431. PubMed PMID: 36388973; PubMed Central PMCID: PMCPMC9646960.

4. Covarrubias AJ, Kale A, Perrone R, Lopez-Dominguez JA, Pisco AO, Kasler HG, et al. Senescent cells promote tissue NAD(+) decline during ageing via the activation of CD38(+) macrophages. Nat Metab. 2020;2(11):1265-83. Epub 20201116. doi: 10.1038/s42255-020-00305-3. PubMed PMID: 33199924; PubMed Central PMCID: PMCPMC7908681.

5. Chini CCS, Peclat TR, Warner GM, Kashyap S, Espindola-Netto JM, de Oliveira GC, et al. CD38 ecto-enzyme in immune cells is induced during aging and regulates NAD(+) and NMN levels. Nat Metab. 2020;2(11):1284-304. Epub 20201116. doi: 10.1038/s42255-020-00298-z. PubMed PMID: 33199925; PubMed Central PMCID: PMCPMC8752031.

6. Kovács KJ. Invited review c-Fos as a transcription factor: a stressful (re)view from a functional map. Neurochemistry International. 1998;33(4):287-97. doi: 10.1016/s0197-0186(98)00023-0.

7. Cabezas-Wallscheid N, Buettner F, Sommerkamp P, Klimmeck D, Ladel L, Thalheimer FB, et al. Vitamin A-Retinoic Acid Signaling Regulates Hematopoietic Stem Cell Dormancy. Cell. 2017;169(5):807-23 e19. Epub 2017/05/10. doi: 10.1016/j.cell.2017.04.018. PubMed PMID: 28479188.

8. Wu X, Dao Thi VL, Huang Y, Billerbeck E, Saha D, Hoffmann HH, et al. Intrinsic Immunity Shapes Viral Resistance of Stem Cells. Cell. 2018;172(3):423-38 e25. Epub 2017/12/19. doi: 10.1016/j.cell.2017.11.018. PubMed PMID: 29249360; PubMed Central PMCID: PMCPMC5786493.

9. Yamashita M, Passegue E. TNF-alpha Coordinates Hematopoietic Stem Cell Survival and Myeloid Regeneration. Cell Stem Cell. 2019;25(3):357-72 e7. Epub 20190620. doi: 10.1016/j.stem.2019.05.019. PubMed PMID: 31230859; PubMed Central PMCID: PMCPMC6733032.

10. Kato Y, Iwama A, Tadokoro Y, Shimoda K, Minoguchi M, Akira S, et al. Selective activation of STAT5 unveils its role in stem cell self-renewal in normal and leukemic hematopoiesis. J Exp Med. 2005;202(1):169-79. doi: 10.1084/jem.20042541. PubMed PMID: 15998795; PubMed Central PMCID: PMCPMC2212906.
11. Wang Z, Li G, Tse W, Bunting KD. Conditional deletion of STAT5 in adult mouse hematopoietic stem cells causes loss of quiescence and permits efficient nonablative stem cell replacement. Blood. 2009;113(20):4856-65. Epub 20090303. doi: 10.1182/blood-2008-09-181107. PubMed PMID: 19258595; PubMed Central PMCID: PMCPMC2686137.
12. Patel B, Zhou Y, Babcock RL, Ma F, Zal MA, Kumar D, et al. STAT3 protects HSCs from intrinsic interferon signaling and loss of long-term blood-forming activity. bioRxiv. 2023. Epub 20230211. doi: 10.1101/2023.02.10.528069. PubMed PMID: 36798265; PubMed Central PMCID: PMCPMC9934695.
13. Luchsinger LL, Strikoudis A, Danzl NM, Bush EC, Finlayson MO, Satwani P, et al. Harnessing Hematopoietic Stem Cell Low Intracellular Calcium Improves Their Maintenance In Vitro. Cell Stem Cell. 2019;25(2):225-40 e7. Epub 20190606. doi: 10.1016/j.stem.2019.05.002. PubMed PMID: 31178255; PubMed Central PMCID: PMCPMC7649753.
14. Morita Y, Ema H, Nakauchi H. Heterogeneity and hierarchy within the most primitive hematopoietic stem cell compartment. J Exp Med. 2010;207(6):1173-82. Epub 2010/04/28. doi: 10.1084/jem.20091318. PubMed PMID: 20421392; PubMed Central PMCID: PMCPMC2882827.
15. Fukushima T, Tanaka Y, Hamey FK, Chang CH, Oki T, Asada S, et al. Discrimination of Dormant and Active Hematopoietic Stem Cells by G0 Marker Reveals Dormancy Regulation by Cytoplasmic Calcium. Cell Rep. 2019;29(12):4144-58 e7. Epub 2019/12/19. doi: 10.1016/j.celrep.2019.11.061. PubMed PMID: 31851939.
16. Sugimura R, He XC, Venkatraman A, Arai F, Box A, Semerad C, et al. Noncanonical Wnt signaling maintains hematopoietic stem cells in the niche. Cell. 2012;150(2):351-65. doi: 10.1016/j.cell.2012.05.041. PubMed PMID: 22817897; PubMed Central PMCID: PMCPMC4492542.
17. Umemoto T, Hashimoto M, Matsumura T, Nakamura-Ishizu A, Suda T. Ca(2+)-mitochondria axis drives cell division in hematopoietic stem cells. J Exp Med. 2018;215(8):2097-113. Epub 20180626. doi: 10.1084/jem.20180421. PubMed PMID: 29946000; PubMed Central PMCID: PMCPMC6080917.
18. Bogeska R, Mikecin AM, Kaschutnig P, Fawaz M, Buchler-Schaff M, Le D, et al. Inflammatory exposure drives long-lived impairment of hematopoietic stem cell self-renewal activity and accelerated aging. Cell Stem Cell. 2022;29(8):1273-84 e8. Epub 20220719. doi: 10.1016/j.stem.2022.06.012. PubMed PMID: 35858618; PubMed Central PMCID: PMCPMC9357150.
19. Matsumoto A, Takeishi S, Kanie T, Susaki E, Onoyama I, Tateishi Y, et al. p57 Is Required for Quiescence and Maintenance of Adult Hematopoietic Stem Cells. Cell Stem Cell. 2011;9(3):262-71. doi: 10.1016/j.stem.2011.06.014.
